# 

**Published:** 1895-03

**Authors:** 


					﻿Vol XV.
MARCH, 1895.
No. 3.
THE
HOMEOPATHIC PHY^ICIAJS,
A Monthly Journal of Homoeopathic Materia Medica
and Clinical Medicine.
“ Be it a freeman whom truth makes free, and all are slaves beside.”
"Seek the Truth: come whence it may, cost what it will.”
EDITED Jim PUBLISHED BY
WALTER M. JANIES, N4. E>.
PHILADELPHIA :
JSTo. 1231 LOCUST STREET.
x. p asf x5 p iT :
ALFRED HEATH A CO., Sons Agknts for Great Britain,
114 Ebury Street, Eaton Square.
4E^Fea**ty Sarfiaertption, $2.50, invariably in advance. Single numbers, 25 ets.
Entered at tbe Philadelphia Peal-Office a* Seooud-Claaa Mail Hatter.*
				

